# Trastuzumab Induced Chemobrain, Atorvastatin Rescued Chemobrain with Enhanced Anticancer Effect and without Hair Loss-Side Effect

**DOI:** 10.3390/jcm8020234

**Published:** 2019-02-11

**Authors:** Seonhwa Lee, Hae-June Lee, Hyunji Kang, Eun-Ho Kim, Young-Cheol Lim, Hyejin Park, Sang Moo Lim, Yong Jin Lee, Jung Min Kim, Jin Su Kim

**Affiliations:** 1 Division of RI Application, Korea Institute of Radiological and Medical Sciences, 75 Nowon-ro, Nowon-gu, Seoul 01812, Korea; remnant8724@naver.com (S.L.); hyunji.k0618@gmail.com (H.K.); zerofe0701@gmail.com (Y.-C.L.); yjlee@kirams.re.kr (Y.J.L.); 2 Department of Bio-convergence Engineering, Korea University, Seoul 02856, Korea; minbogun@korea.ac.kr; 3 Division of Radiation Biomedical Research, Korea Institute of Radiological and Medical Sciences, 75 Nowon-ro, Nowon-gu, Seoul 01812, Korea; hjlee@kirams.re.kr (H.-J.L.); eh140149@kirams.re.kr (E.-H.K.); jhp13@daum.net (H.P.); 4 Radiological and Medico-Oncological Sciences, University of Science and Technology, 75 Nowon-ro, Nowon-gu, Seoul 01812, Korea; 5 Research Support Team, ANDIVA Inc., Chuncheon 24324, Korea; 6 Department of Nuclear Medicine, Korea Institute Radiological and Medical Sciences, 75 Nowon-ro, Nowon-gu, Seoul 01812, Korea; smlim328@kcch.re.kr

**Keywords:** chemo-brain, trastuzumab therapy, atorvastatin, anti-cancer effect, radiomics

## Abstract

The authors identified that chemo-brain was induced after trastuzumab (TZB) therapy. In addition, atorvastatin (ATV) could rescue chemo-brain during trastuzumab (TZB) therapy. Enhanced therapeutic effect of TZB was confirmed after ATV therapy. We also investigated that there was no hair loss side effect due to ATV therapy. In an animal model, 150 μg TZB and five serial doses of 20 mg/kg ATV were administered. ^18^F-fluorodeoxyglucose Positron Emission Tomography (PET) and Magnetic Resonance Imaging (MRI) data were acquired. Statistical parametric mapping analysis and voxel-based morphometry analysis were performed to identify differences in glucose metabolism and gray matter concentration. The enhanced therapeutic efficacy of TZB after ATV treatment was assessed using a human epidermal growth factor receptor 2-positive gastric cancer model. We found a decrease in cerebral glucose metabolism and gray matter concentration in the frontal lobe following TZB therapy (*p* < 0.005). After subsequent ATV administration, glucose metabolism and regional gray matter concentration were rescued (*p* < 0.005). Cognitive impairment due to TZB and the rescue effect of ATV were confirmed using a passive avoidance test and quantitative real-time reverse transcription PCR. Furthermore, the penetration and accumulation of TZB in tumors increased by 100% after ATV co-administration, which resulted in an enhanced anti-cancer effect. Our study collectively demonstrates that ATV co-administration with TZB rescued the TZB-induced chemo-brain and enhances the therapeutic efficacy of TZB in tumors. We also showed that there was no hair loss during ATV therapy.

## 1. Introduction

Human epidermal growth factor receptor 2 (HER2) is a tyrosine kinase receptor involved in the pathogenesis of several cancers, including advanced gastric and gastroesophageal junction cancer [1]. HER2 protein binds to an extracellular ligand binding domain that initiates a signal transduction cascade that affects tumor cell biology through several mechanisms, including cell proliferation, apoptosis, adhesion, migration, and differentiation [2]. Trastuzumab (TZB) (Roche, Boston, MA, USA) is the first humanized monoclonal antibody (mAb) approved for immunotherapy and the first oncogene-targeted treatment with a proven survival benefit in HER2-positive cancer [3]. Several studies have examined the efficacy of anti-HER2 therapies in both in vitro and in vivo models of gastric cancer using TZB and have shown that TZB leads to decreased HER2 signaling and inhibits cell growth [4]. Although the clinical benefit of TZB was well described, it may be associated with cardio toxicity, which can lead to left ventricular dysfunction and congestive heart failure [5]. Until now, there was no report regarding chemo-brain after TZB therapy.

Statins, which are well-known inhibitors of HMG-CoA reductase in cholesterol synthesis, are widely used to lower cholesterol for the treatment of hypercholesterolemia [6,7]. Recently, they have been reported to exhibit anti-tumor activities in various cancer cells mediated by anti-proliferative, proapoptotic, and anti-invasive properties [8,9,10]. Atorvastatin (ATV) was reported to promote cell death and radio-sensitivity in prostate cancer cells [11]. The effect of attenuating cognitive impairment after ATV therapy was also reported in a cellular level and mouse mode [12,13]. In addition, Taylor BA et al. reported that neural activity and cognition function improved after administration of ATV by f-MRI study in humans [14].

Chemotherapy-associated cognitive impairment was referred to as chemo-brain. Although several candidate mechanisms explain chemo-brain, the accurate biological pathway remains obscure [15,16]. However, studies from humans and animal models have been published that focus on the relation between inflammatory markers, as a proxy for neuroinflammation, and cognitive performance. And many studies suggest that cognitive dysfunction may involve the actions of cytokines including IL-6, IL-1β, and TNF-α and microglia activation [17,18,19]. Previously, oneauthor in this study reported donepezil, which is a reversible acetylcholinesterase inhibitor, improves cognitive impairment during doxorubicin or cyclophosphamide therapy [20]. In this study, we investigated cognitive impairment after administration of TZB, which has not yet been explored. We also showed that administration of ATV (Pfizer, New York, NY, USA) could compensate for cognitive impairment resulting from TZB therapy. Cognitive impairment was assessed by measuring cerebral glucose metabolism using statistical parametric mapping (SPM) analysis, regional gray matter concentration using voxel-based morphometry (VBM) analysis, the passive avoidance test, and quantitative real-time reverse transcription polymerase chain reaction. Alongside the enhanced anti-cancer effect of TZB, administration of ATV was confirmed by an ex vivo radiomics analysis, in vitro cell viability, immunoblotting of cell lysates, and in vivo measurements of tumor size. We also investigated that there was no hair loss side effect due to ATV therapy although ATV has anti-tumor activities for the NCI-N87 HER2 positive tumor mouse model.

## 2. Materials and Methods

### 2.1. Animals

In this study, five-week-old female BALB/C nude mice and C57BL/6 mice were obtained from Shizuoka Laboratory Center (Japan). Mice were maintained in temperature-controlled clean racks with a 12-h light/dark cycle. The mice were allowed to acclimatize for one week before the start of the experiment. All experiments were performed in accordance with the institutional guidelines of the Korea Institute of Radiological & Medical Sciences. BALB/nude mice were used for cognitive impairment and anti-tumor studies. C57BL/6 mice were used for alopecia studies.

### 2.2. Assessment of Chemobrain Using PET SPM and MR VBM

#### 2.2.1. Cognitive Deficit Model

**Figure 1:**
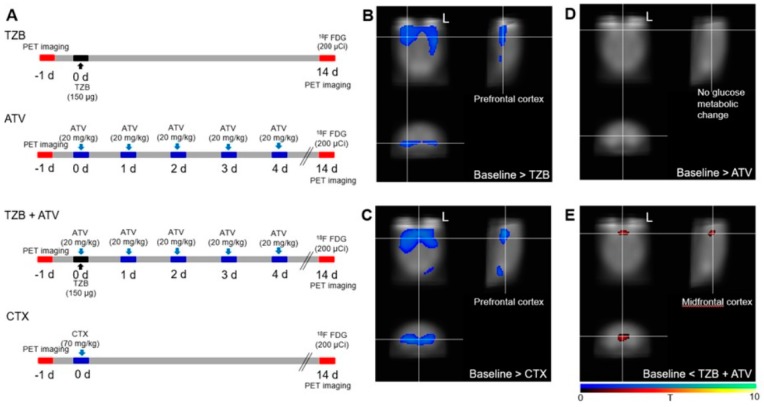
PET evidence of ATV effect during TZB therapy. (**A**) The experimental design. (**B**) A decrease in cerebral glucose metabolism was observed in the region of the bilateral frontal lobe following administration of TZB or (**C**) CTX relative to baseline (*p* < 0.005). (**D**) No significant difference was found between ATV treatment and baseline (*p* < 0.005). (**E**) An increase in cerebral glucose metabolism was observed in the region of the medial prefrontal cortex following administration of ATV (*p* < 0.005). Decreased glucose metabolism after TZB treatment was rescued after administration of ATV. PET, positron emission imaging. FDG PET, ^18^F-fluorodeoxyglucose positron emission tomography. ATV, atorvastatin. TZB, trastuzumab. CTX, cyclophosphamide. (*n* = 10 per group).

**Figure 2:**
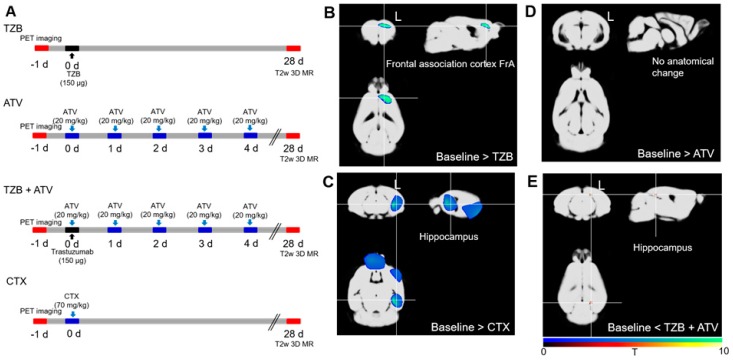
MR VBM evidence of ATV effect during TZB therapy. (**A**) The experimental design. (**B**) A decrease in gray matter concentration was observed in the region of the left frontal association cortex during TZB therapy and in the region of the frontal association cortex and hippocampus on the left side. (**C**) During CTX therapy relative to the baseline (*p* < 0.005). (**D**) No significant difference between ATV and the baseline was observed (*p* < 0.005). (**E**) Decreased regional gray matter concentration in the region of the frontal association cortex during TZB therapy was rescued when ATV was administered (*p* < 0.005). MR, magnetic resonance imaging. T2w, T2-weighted imaging. 3D, three-dimensional. VBM, voxel-based morphometry. ATV, atorvastatin. TZB, trastuzumab. CTX, cyclophosphamide. (*n* = 7 per group).

A cognitive deficit was evaluated after administration of 150 μg of TZB (*n* = 7 per group, female). A total of 20 mg/kg of ATV was administered for 5 days to determine any effects on recovery of the cognitive deficit. Similarly, 70 mg/kg (equivalent to a dose of 215 mg/m^2^ in humans, which is within the range of doses typically used for treatment of women with metastatic breast cancer (100–250 mg/m^2^)) of cyclophosphamide (CTX) was administered. The experimental design, including the drug doses, dose schedule, and PET imaging schedule used, is described in Figure 1A, and the MR imaging schedule is described in Figure 2A.

#### 2.2.2. PET Data Acquisition

Siemens Inveon PET was used in this study [21]. Regional cerebral glucose metabolism was measured using ^18^F-fluorodeoxyglucose (FDG) PET. Before PET scanning, mice (*n* = 10 per group, female) fasted for at least 8 h, after which they were anesthetized with 2% isoflurane in 100% oxygen (Forane solution, Choongwae Pharma, Seoul, Korea). During PET scanning, the body temperature was maintained at 36 °C with heating pads. Then, 200 μCi of ^18^F-FDG was injected through a tail vein. After 30 min of uptake, 30-min emission PET data were acquired with an energy window of 350–650 keV. Emission list-mode data were sorted into three-dimensional (3D) sinograms and reconstructed using 3D reprojection algorithms. No filter was applied. The image matrix measured 256 × 256 × 159, the pixel size was 0.155 × 0.155 mm^2^, and the slice thickness was 0.796 mm.

#### 2.2.3. Voxel-Based Statistical Analysis of PET Data

Voxel-wise statistical analysis was performed to identify regional differences between groups using SPM 8 (http://www.fil.ion.ucl.ac.uk/spm). SPM analysis for small animals was described in our previous study [22]. For SPM analysis, the brain was extracted and a study-specific mouse brain template was constructed. Individual PET data were spatially normalized onto the mouse brain template. Spatial normalization for individual PET was performed using affine and nonlinear transformations. The voxel size of spatially normalized images was 0.3 × 0.3 × 0.3 mm^3^. Additionally, a 3-mm Gaussian smoothing kernel was applied to enhance the signal-to-noise ratio. Count normalization was performed. Paired *t*-tests were used to identify regional differences in cerebral glucose metabolism between groups (*p* < 0.005, uncorrected).

#### 2.2.4. MR Data Acquisition

T2w 3D MR images were acquired using an Agilent 9.4 T MR scanner (USA). An AD quad 70 RF coil was used, and the matrix size was 192 × 192 × 192. The repetition time (RT) was 2500 ms. The effective echo time (TE) was 7.00 ms, and the image acquisition time was 3 h 36 min.

#### 2.2.5. VBM Analysis of MR Data

Modulated Voxel-based morphometry (VBM) was performed to compare the regional gray matter concentration between groups in particular brain regions [23]. Skull stripping was performed using BrainSuite (version 16) software [24]. Parameters including brain surface extractor diffusion iterations, diffusion constant, edge constant, and erosion size were adjusted for skull stripping using individual T2w 3D MR data. Skull-stripped MR data were set to dimensions of 512 × 512 × 512 with a voxel size of 0.04 × 0.04 × 0.04 mm^3^. A predefined gray matter template created by the Delora research team was used for spatial normalization [25]. Individual skull-stripped MR data were spatially normalized onto the template and smoothed with a 2-mm Gaussian kernel. SPM 8 was used for VBM analysis. Paired *t*-tests were used to identify regional differences in gray matter concentrations between groups (*p* < 0.005, uncorrected).

### 2.3. Behavioral Analysis and Biomarker of Cognitive Deficit Model

#### 2.3.1. Passive Avoidance Test

**Figure 3:**
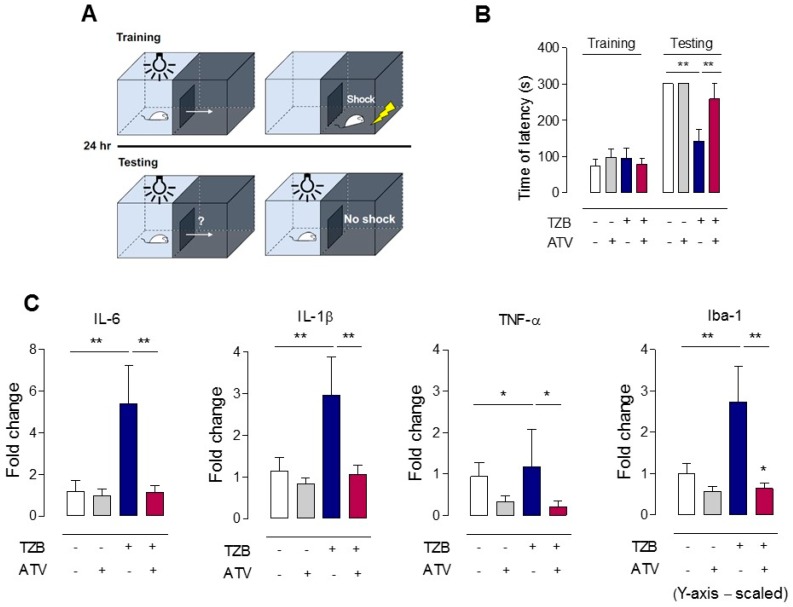
Behavioral study and pertaining the level of cytokines. (**A**) Schematic of passive avoidance test. Passive avoidance tests were performed to evaluate memory impairment in all groups. (**B**) The mean time of latency. The ATV co-administration group significantly extended the mean time of step-through latency (time of latency was 259 s) compared with that of the single TZB administration group. Values are means ± standard deviation of the mean (*n* = 6). (** *p* < 0.005). (**C**) Quantitative real-time reverse transcription polymerase chain reaction. (* *p* < 0.05 and ** *p* < 0.005). ATV, atorvastatin. TZB, trastuzumab.

The passive avoidance test was used to assess the response to an aversive stimulus and long-term memory. The apparatus consisted of two divided rooms and a grilled floor for electric stimulation. A steel board separated the two rooms and could automatically be moved up and down. In the adaptation session, mice traveled freely between the two rooms for 5 min. The next day, the rooms were divided, and each mouse was placed in one room. The room was kept dark for 60 s to allow the mouse to adjust to darkness, and then the light was turned on as the steel panel was moved simultaneously. As the mouse traveled across to the other room to avoid the bright light, the mouse’s movement caused the steel panel to shut, and an electrical shock impulse (0.15 mA, 2 s) was transmitted to the grill. After 24 h, the mice were subjected to the same trial without electrical shock, and the latency time to cross over to the other room was automatically recorded (Figure 3A).

#### 2.3.2. Quantitative Real-Time Reverse Transcription PCR (qPCR)

Total RNA was extracted from the mouse frontal cortex using QIAzol Lysis Reagent (QIGEN, Hilden, Germany). cDNA was synthesized by amfiRivert cDNA synthesis platinum master mix (GenDEPOT, Katy, TX, USA). Real-time quantitative PCR analysis was performed using a qPCR Green 2X Master Mix kit (m.biotech, Gyeonggi-do, Korea) and a LightCycler^®^ 96 Instrument (Roche Molecular Systems, Basel, Switzerland). The PCR protocol was as follows: initial denaturation for 10 min at 95 °C, 42 cycles of 95 °C for 15 s, 58 °C for 20 s, and 72 °C for 30 s. The comparative Ct method was used to determine the normalized changes in the target gene relative to a calibrator reference (Gapdh). The sequence-specific primers were as follows: mouse IL6 forward 5′-CCTTCCCTACTTCACAAGTC-3′, reverse 5′-TTTTCTGCAAGTGCATCATC-3′, and mouse Il-1β forward 5′-ACCTTTTGACAGTGATGAGAA-3′, reverse 5′-GCTGCTGCGAGATTTGA-3′, TNF-α forward 5′-TGGGTTGTACCTTGTCTACT-3′, reverse 5′-TGGTATGAGATAGCAAATCGG-3′, mouse Iba1 forward 5′-CCTTCCCTACTTCACAAGTC-3′, 5′-TTTTCTGCAAGTGCATCATC-3′, Gapdh forward 5′-CAAGAAGGTGGTGAAGCAGG-3′, and reverse 5′-AGGTGGAAGAGTGGGAGTTG-3′.

#### 2.3.3. Assessment of Abnormal Hair Regrowth Using the Alopecia Model

**Figure 4:**
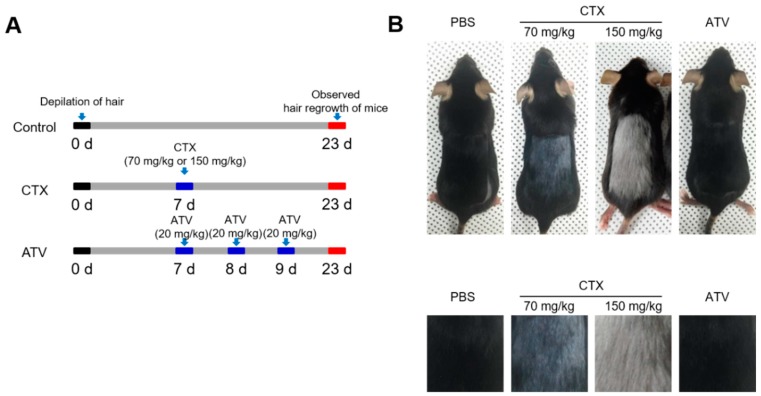
Evidence of no side effect of ATV on hair regrowth. (**A**) The design of the experiment performed to test the in vivo side effect of ATV on hair regrowth in C57BL/6 mice. (**B**) Representative images of back skin gloss of mice and cropped image with higher magnification. ATV, atorvastatin. PBS, phosphate-buffered saline. CTX, cyclophosphamide. (*n* = 7 per group).

To investigate whether ATV induced abnormal hair regrowth in mice, hair was shaved from the dorsal surface of the back in C57BL/6 mice (*n* = 7 per group) by shaving and waxing using wax strips (Veet, Slough, UK), and the skin was washed using PBS. Mice were administered PBS, CTX, and ATV (all I.P.). After 23 days from depilation of hair, hair growth and hair color were observed. The doses and dose schedule was described in detail in Figure 4A.

### 2.4. Enhanced Anti-Cancer Effect of Trastuzumab after Administration of Atorvastatin

#### 2.4.1. Cell Culture and Tumor Xenograft in Mice

NCI-N87 HER2-positive cancer cell lines obtained from the American Type Culture Collection were maintained in RPMI containing 10% fetal bovine serum with antibiotics (Sigma, Darmstadt, Germany) at 37 °C in a humidified 5% CO_2_ incubator. Cells were seeded at a density of 20,000 cells per well in 96-well plate in four replicates. After 24 h, the medium was replaced with a new medium containing ATV and/or TZB. Following 48 h of incubation, alamarBlue reagent (Bio Rad, Hercules, CA, USA) was added directly to cells and incubated for 2 hours at 37 °C. NCI-N87 cells (5 × 10^6^) were subcutaneously injected into female BALB/nude mice (*n* = 5 per group). Tumor size was measured using a digital caliper and tumor volume was determined using the formula width^2^ × length × 0.4.

#### 2.4.2. Labelling and Purification of Alexa-488-TZB

A solution of Alexa-Fluor-488 NHS Ester (Invitrogen, Waltham, MA, USA) was prepared in dimethyl sulfoxide with 1% acetic acid. This solution was immediately dissolved in 500 μL (10 mg/mL) of 1 M sodium bicarbonate solution, which had a pH of 8.4. The solution was mixed thoroughly and allowed to stand for 1 h at room temperature. This reaction solution was purified with a size exclusion PD-10 column (GE Healthcare Bio-Sciences AB, Uppsala, Sweden) with PBS as the elution buffer. An aliquot (100 μg/100 μL PBS, pH 7.2) of the purified Alexa-488-TZB and unconjugated purified solution were measured using a NanoDrop spectrophotometer (ThermoFisher Scientific, Waltham, MA, USA). The number of Alexa-488 molecules conjugated per unit TZB was estimated by the Alexa-488 peak intensity distributed between the mAb-Alexa-488 conjugate and free Alexa-488 eluted from size-exclusion high-performance liquid chromatography. This reaction resulted in Alexa-488-TZB with 2.6 Alexa-Fluor-488 molecules per TZB. The concentration of Alexa-488-TZB was 1.5 mg/mL.

#### 2.4.3. Immunofluorescence Staining of Tumor Tissues

**Figure 5:**
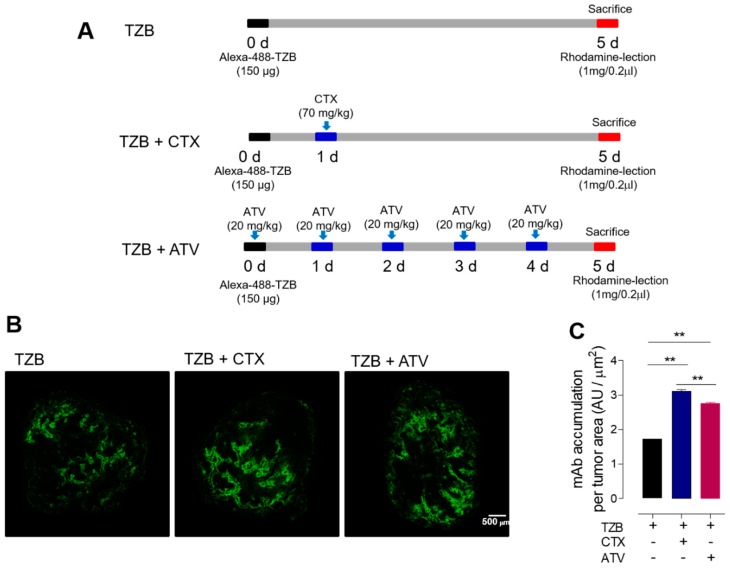

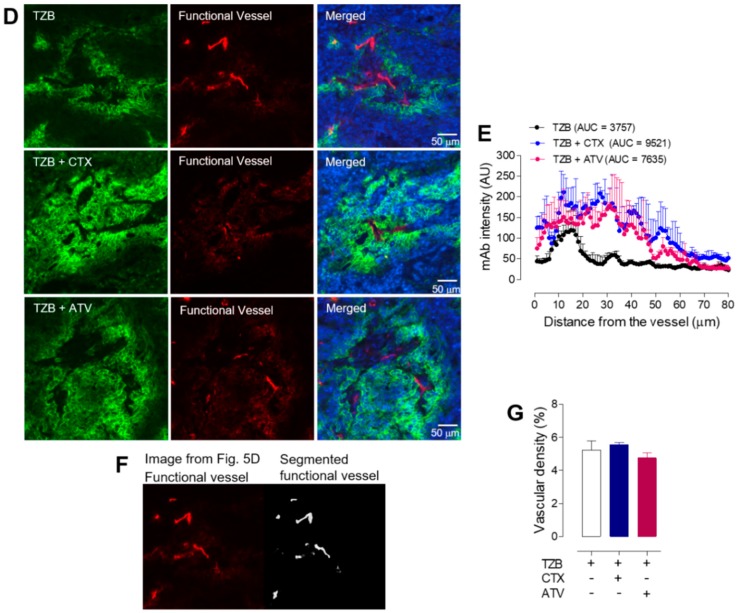
Effect of ATV on TZB accumulation in the tumor tissue. (**A**) The experimental design of TZB penetration into tumors. (**B**) In vivo localization of Alexa-488-TZB in NCI-N87 tumor tissue (scale bar = 500 μm). (**C**) Alexa-488-TZB accumulation per tumor tissue area (** *p* < 0.005), (**D**) In vivo penetration of Alexa-488-TZB after co-administration with ATV in NCI-N87 tumor tissue. Representative images of Alexa-488-TZB (green), a rhodamine-lectin stained functional blood vessel (red), and green and red merged with DAPI staining (blue) (scale bar = 50 μm). (**E**) In vivo penetration of Alexa-488-TZB is represented by histograms of Alexa-488 fluorescent intensity. (**F**) Images of functional vessel and segmented functional vessel. (**G**) Density of rhodamine-lectin positive functional blood vessel (N.S.). ATV, atorvastatin. TZB, trastuzumab. CTX, cyclophosphamide. (*n* = 7 per group).

When the tumor size reached 200 ± 20 mm^3^, Alexa-488-TZB was I.V. injected as a single dose (150 μg) in the control group (*n* = 7). In the ATV-treated group (*n* = 7), 20 mg/kg of ATV in a 0.2 mL volume was I.P. injected for 5 days [26]. To compare the therapeutic effect of ATV, CTX (70 mg/kg) was I.P. injected in another group of animals (*n* = 7) [27]. Five days later, the mice received a lateral tail vein injection of rhodamine-lectin (Rhodamine Ricinus communis Agglutinin I, 1 mg in 0.2 mL of PBS) to delineate blood vessels, and, 5 min after the lectin injection, the mice were euthanized by CO_2_ inhalation and exsanguinated by cardiac puncture before dissection. The doses and dose schedule are described in detail in Figure 5A.

Tumors were harvested and flash-frozen with the skin intact. Tumors were fixed with 4% paraformaldehyde overnight at 4 °C and were then cryopreserved with 30% sucrose in PBS until the tissue sank to the bottom of the tube at 4 °C. Tumors were embedded in an optimal cutting temperature compound for 1 h at −20 °C prior to sectioning and stored at −70 °C until use. Tumors were sectioned using a Leica CM 1850 cryostat (Leica Microsystems, Buffalo Grove, IL, USA) to a thickness of 8 µm to cover the entire tumor tissue. The 8-µm-thick cryostat sections were mounted on SuperFrost Plus slides. Before staining, slides were warmed at room temperature for 10 min. Slides were rehydrated with 200 µL of PBS for 10 min and then fixed in 4% paraformaldehyde for 10 min. Lastly, the DAPI stain was applied, and slides were cover-slipped.

#### 2.4.4. Acquisition and Analysis of Fluorescent Images

Fluorescent images were acquired with a 10× objective using a fluorescence microscope (In cell analyzer 2200, GE Healthcare, Milwaukee, NY, USA) equipped with mosaic stitching software (In cell developer toolbox, GE Healthcare, Milwaukee, NY, USA). Three independent channels were obtained as follows: DAPI for nuclei (shown in blue, Ex/Em = 358/461 nm), rhodamine for blood vessels (shown in red, Ex/Em = 558/581 nm), and FITC for Alexa 488-TZB (shown in green, Ex/Em = 488/525 nm). Values were grouped together from 12 regions in each tumor. Four regions were measured 25% of the distance from the tumor apex, four regions were measured from the central region of the tumor, and four regions were measured 75% of the distance from the tumor apex. Each tumor was treated as an independent sample (*n* = 5).

#### 2.4.5. Measurement of Alexa-488-TZB Accumulation in Tumor Tissues

Image analysis was performed using an in-house program written in MATLAB (MathWorks, Natick, MA, USA). Individual image channels were exported as TIFF images for analysis. For the measurement of Alexa-488-TZB penetration from the vessel and tumor surface, a region of interest (ROI) was drawn to calculate the intensity of Alexa-488-TZB. The penetration of Alexa-488-TZB up to a depth of ~80 µm depth from the vessel and tumor surface was calculated by area under the curve analysis. The total accumulation of Alexa-488-TZB was calculated by total Alexa-488-TZB intensity in the tumor divided by the total tumor area.

#### 2.4.6. Microvascular Analysis in Tumor Tissues

Overall micro-distribution of functional vessels was assessed by measuring vascular density. To calculate vascular density, an individual blood vessel image was segmented using the fuzzy c-means clustering method. A value of 1 was regarded as the vessel area and 0 was regarded as the non-vessel area on a segmented binary image. The fraction of vessel area over the entire tumor area (vessel area/non-vessel area) was represented as vascular density. Microvascular analysis was performed using the in-house MATLAB code and the MIPAV program (National Institutes of Health, Bethesda, MD, USA).

#### 2.4.7. Radiomics Analysis of the Cell Nuclei in Tumor Tissues

Radiomics analyses such as circularity and fractal dimension (FD) analyses for cell nuclei were performed to identify morphological changes after treatment in each group. Measurements of circularity or FD for cell nuclei demonstrate the therapeutic effect of a drug [28]. Circularity and FD analyses for cell nuclei in tumor tissues were performed near the vessel (~80 µm from the vessel). The edge contour information of DAPI cell nuclei was extracted using a Laplacian edge-finding algorithm. Circularity and FD analysis were then performed. Image analysis was performed using the in-house MATLAB code and the MIPAV program (National Institutes of Health, Bethesda, MD, USA).

### 2.5. Measurement of Tumor Growth after Co-Administration of ATV

#### Cell Viability Assay, Cell Lysate Analysis, and Tumor Growth Delay

Cell viability was measured at an emission wavelength of 590 nm using a microplate reader. We examined whether ATV and/or TZB affects NCI-N87 cell proliferation following treatment by counting cells. Various concentrations (0, 1, 5, 10, 50, 100, 200, 300, 400, and 500 μM) of ATV and (0, 1, and 5 μg/mL) of TZB were used. Next, cytotoxicity resulting from the poly (ADP-ribose) polymerase 1 and Bcl-2, which are a unique characteristic of apoptotic cell death, was proven by immunoblotting cell lysates using Anti-Bcl-2 antibody (cat No. ab59348) (Abcam, Rockville, MD, USA) and Anti-PARP1 antibody (cat No. ab32138) (Abcam, Rockville, MD, USA).

When the tumor volume in NCI-N87-bearing mice was 200 ± 20 mm^2^, the mice were randomly divided into five groups (*n* = 3–5 per group). Each group was treated with TZB (150 μg) or TZB (150 μg) plus ATV (20 mg/kg for 5 days). The tumor size was measured using a digital caliper, and the tumor volume was calculated three times a week, according to the following formula: width^2^ × length × 0.4.

### 2.6. Statistics

Data are expressed as means ± standard deviation. The differences between groups were analyzed using a one-way analysis of variance. PRISM 5 (GraphPad software, San Diego, CA, USA) was used for statistical analysis.

### 2.7. Study Approval

All applicable international, national, and/or institutional guidelines for the care and use of animals were followed. The animal study was approved by the Institutional Animal Care and Use Committee (IACUC) and Institutional review board in Korea Institute of Radiological and Medical Sciences (#KIRAMS 2018-0016).

## 3. Results

### 3.1. Evidence of Statistical Parametric Mapping (SPM) of PET and Optimized Voxel Based Morphometry (VBM) of MR

#### 3.1.1. Trastuzumab Therapy Induced Chemo-Brain

A decrease in cerebral glucose metabolism was observed in the region of the bilateral frontal lobe after administration of TZB or CTX compared with the baseline level (Figure 1B,C, *p* < 0.005).

There was a decrease in gray matter concentration in the region of the frontal association cortex on the left side following TZB therapy, and in the region of the frontal association cortex and hippocampus on the left side following CTX therapy, relative to baseline (Figure 2B,C, *p* < 0.005).

#### 3.1.2. Atorvastatin Has No Chemo-Brain Effect

There was no significant difference in cerebral glucose metabolism between the ATV-treated group and baseline (Figure 1D, *p* < 0.005). There was no significant difference in gray matter concentration between the ATV-treated group and baseline (Figure 2D, *p* < 0.005). This result demonstrated that there was no chemo-brain effect during ATV therapy.

#### 3.1.3. Atorvastatin Rescued Cognitive Impairment during Trastuzumab Therapy

Decreased glucose metabolism after TZB therapy was rescued by co-administration of ATV. An increase in cerebral glucose metabolism was noted in the medial prefrontal cortex after co-administration of ATV (Figure 1E, *p* < 0.005).

In addition, we observed the decrease in gray matter concentration in the frontal association cortex resulting from TZB therapy, which was rescued when ATV was co-administered (Figure 2E, *p* < 0.005). This result demonstrated that ATV could rescue the chemo-brain effect during TZB therapy.

### 3.2. Evidence of Passive Avoidance Memory Test

#### 3.2.1. Atorvastatin Rescued Cognitive Impairment during Trastuzumab Therapy

To examine the effect of ATV on TZB-induced memory impairment, mice underwent a passive avoidance memory test (Figure 3A). The schedule of administration of TZB and ATV was the same as that for PET imaging shown in Figure 1A. Twenty-four hours after the training sessions, memory function was not affected upon administration of ATV alone, but TZB exhibited significant memory impairment (the mean time of latency, 300 s in control vs. 143 s in TZB alone, ** *p* < 0.005). ATV co-administration significantly extended the mean time of step-through latency (time of latency was 259 s, ** *p* < 0.005) compared with that achieved by single TZB administration (Figure 3B).

#### 3.2.2. Evidence of IL-6, IL-1β, and TNF-α Levels

Figure 3C shows the results pertaining to the levels of the cytokines IL-6, IL-1β, and TNF-α. Because PET imaging showed significant changes in the cerebral glucose metabolism of the frontal lobe with administration of TZB or ATV, we examined the extent of inflammation by measuring pro-inflammatory markers in the frontal cortex. The mRNA expressions IL-6, IL-1β, and TNF-α were assessed 14 days after TZB administration. TZB caused a significant increase in IL-6 and IL-1β levels, which were down-regulated by ATV treatment (** *p* < 0.005). TNF-α. expression was decreased with the administration of ATV alone and with co-administration compared with the control level (* *p* < 0.05). We also observed significant alteration of Iba1 (** *p* < 0.005), which is a marker of microglial activation representing neuro-inflammation as well.

#### 3.2.3. Atorvastatin Has No Abnormal Hair Regrowth Side Effect

Figure 4B shows the results of hair loss in C57BL/6 mice. The shaved skin of telogen mice was pink and darkened with anagen initiation. After 23 days, normal black hair was observed in PBS-treated and ATV-treated mice while abnormal gray and white hair was observed in CTX-treated mice receiving either 70 mg/kg or 150 mg/kg of the drug. Gross morphological assessment revealed that ATV treatment did not appear to prompt abnormal hair regrowth in mice, in contrast to those receiving CTX.

#### 3.2.4. Atorvastatin Enhanced Anti-Cancer Effect during Trastuzumab Therapy

Figure 5B shows the accumulation of Alexa 488-TZB in NCI-N87 tumor tissue. The level of Alexa-488-TZB accumulation was 1.72 ± 0.01 AU/µm^2^ in the group receiving Alexa-488-TZB alone, 3.10 ± 0.05 AU/µm^2^ in the group receiving Alexa-488-TZB with CTX co-administration, and 2.76 ± 0.02 AU/µm^2^ in the group receiving Alexa-488-TZB with ATV co-administration (Figure 5C). The accumulation of Alexa-488-TZB increased by 80% and 60% when co-administered with CTX and ATV, respectively, compared with the accumulation observed for Alexa-488-TZB alone (** *p* < 0.005). Figure 5D shows the penetration of Alexa-488-TZB from blood vessels. The intensity profile of Alexa-488-TZB with distance from the tumor surface was plotted (Figure 5E). Penetration of Alexa-488-TZB to a distance of 80 µm from the vessel represents an increase of 151% and of 100% during co-administration with CTX or ATV, respectively. In addition, the highest amount of Alexa-488-TZB was shifted toward the tumor core from 15 μm to 27 μm when ATV was co-administered. Figure 5F shows a functional vessel and a segmented functional vessel. Functional vessel density tended to decrease after administration of ATV, but the change was not significantly different. This finding indicates that higher accumulation of Alexa-488-TZB was not due to differences in vascular density among the groups (Figure 5F).

**Figure 6:**
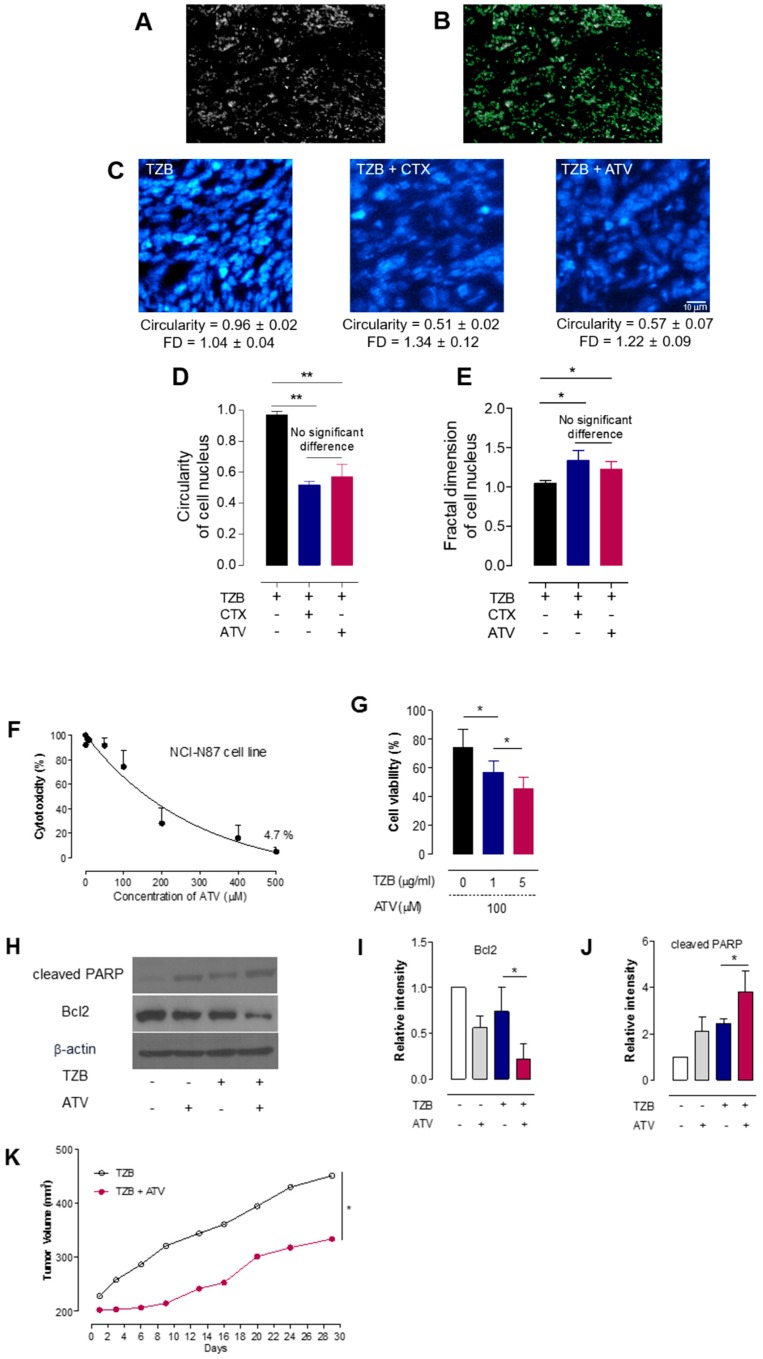
Cytotoxicity of ATV and effect of ATV on apoptosis and tumor growth delay. (**A**) Ex vivo image of cell nuclei. (**B**) The edge contours of DAPI-stained cell nuclei were extracted using a Laplacian edge-finding algorithm. (**C**) Representative images of cell nuclei when CTX or ATV was co-administered with TZB. (**D**,**E**) Circularity and fractal dimension of the cell nucleus in tumor tissue (mean ± standard deviation) (** *p* < 0.005 and * *p* < 0.05). ATV, atorvastatin. TZB, trastuzumab. CTX, cyclophosphamide. FD, fractal dimension. (**F**) Cytotoxicity assay according to the concentration of ATV. (**G**) Cell viability according to the concentration of TZB with ATV (* *p* < 0.05). (**H**) Cell lysates were immunoblotted with the indicated antibodies. (**I**) Relative intensity of Bcl-2. (**J**) Relative intensity of cleaved PARP. (*n* = 3, mean ± standard deviation, * *p* < 0.05). (**K**) Tumor growth delay for 30 days (*n* = 3–5 per group). ATV, atorvastatin. TZB, trastuzumab. PARP, poly (ADP-ribose) polymerase 1.

Figure 6 shows the results of circularity and FD analyses of cell nuclei. Nuclear DNA in healthy cells displays high circularity while cells undergoing apoptosis display low circularity. Therefore, a decrease in circularity was considered evidence of a therapeutic effect. Cell nuclei were fragmented and damaged following the TZB, ATV, and CTX treatments. Representative Alexa-488-TZB–treated cells displayed high circularity while those undergoing apoptosis displayed low circularity following co-administration of ATV and CTX, which is proximal to the blood vessel (up to an 80-μm distance). The circularity of the cell nuclei was 0.96 ± 0.02 in the TZB-only group, 0.51 ± 0.02 in the CTX co-administration group, and 0.57 ± 0.07 in the ATV co-administration group. In the proximal region, the circularity in both the CTX and ATV co-administration groups was significantly lower than that in the TZB-only group (** *p* < 0.005). The FD of the cell nuclei was 1.04 ± 0.04 in the TZB-only group, 1.34 ± 0.12 in the CTX co-administration group, and 1.22 ± 0.09 in the ATV co-administration group. In the proximal region, the circularity in both the CTX and ATV co-administration groups was significantly higher than that in the TZB-only group (* *p* < 0.05).

Figure 6F showed cytotoxicity induced by ATV. The growth of NCI-N87 cells was dose-dependently decreased by ATV. Figure 6G showed cytotoxicity induced by ATV and various concentrations of TZB. Cytotoxicity was proven by immunoblotting of cell lysates using indicated antibodies. The analysis showed that poly (ADP-ribose) polymerase 1 cleavage was synergistically enhanced by treatment with ATV + TZB compared with treatment with ATV or TZB alone. This finding was further supported by the significant decrease in Bcl-2 expression observed with ATV + TZB (* *p* < 0.05) (shown in Figure 6H–J). Figure 6K showed delayed tumor growth after co-administration of ATV over 30 days. At 30 days after administration of TZB and/or co-administration of ATV, tumor size decreased by 36% after co-administration of ATV (* *p* < 0.05).

## 4. Discussion

### 4.1. Atorvastatin Rescued Cognitive Impairment during Trastuzumab Therapy without Chemo-Brain and Hair-Loss Side Effect

The present study shows that TZB induces cognitive impairment, which is a side effect that occurs in addition to its anti-tumor effects, in a HER2-positive gastric cancer model. The study shows that co-administration of ATV with TZB can rescue the resulting cognitive impairment. In addition, co-administration of ATV with TZB can increase the anti-cancer effect of TZB with no side effect of hair loss.

### 4.2. Why Atorvastatin Therapy Could Rescue the Chemo-Brain Effect during Trastuzumab Therapy

We found that cerebral glucose metabolism and regional gray matter concentration in the region of the prefrontal cortex or frontal association cortex was decreased when TZB was administered. Because TZB cannot penetrate the brain-blood barrier, it cannot act on the brain directly. It has been previously reported that IL-6 is increased after administration of TZB [29]. Therefore, cognitive impairment during TZB therapy may be explained by increased IL-6 levels [30]. In our present study, the degree of cognitive impairment following TZB therapy and the compensatory effects of ATV were confirmed using a passive avoidance test and measuring the levels of IL-6, IL-1β, and TNF-α. According to the VBM results, only the frontal association cortex and left hippocampus were altered. The left hippocampus was highly correlated with an associative spatial long-term memory [31]. Decreased grey matter concentration in the left hippocampus might represent a decrease in associative spatial long-term memory.

The issue of chemo-brain with breast cancer patients was described in our previous study in detail [15]. According to the report, large number of drugs such as rituximab, cyclophosphamide, doxorubicin, vincristine and prednisone, vincristine and rituximab, or vincristine and bendamustine were discussed [15]. However, there was no report on chemo-brain after treatment of TZB. In our present study, we first showed the effect of chemo-brain after treatment of TZB. Additionally, we also showed ATV can rescue the symptom of chemo-brain after treatment of TZB. Recovery of cognitive deficit after use of ATV was also found in the previous study [14] and well matched the result, which was performed from our groups. In our present study, we extensively analyzed the effect of rescue of chemo-brain after ATV treatment using an imaging marker such as FDG PET and Voxel based morphometry, and behavioral study. Cytokine analysis also supported the image analysis.

However, we used nude mice in this study and a nude mouse has a genetic mutant that has a deteriorated or removed thymus, which results in an inhibited immune system due to a greatly reduced number of T cells [32]. In chemotherapy-induced dyscognition, cytokines are presented as an important pathway or as a marker for the prediction. Therefore, these nude mice may have limitations in studying chemo-brain.

### 4.3. Advantage As an Anticancer Drug of Atorvastatint during Trastuzumab Therapy

An advantage of ATV as a combinatorial drug with TZB is the enhanced therapeutic efficacy of TZB in tumors. ATV enhanced accumulation and penetration of TZB in solid tumor tissue in a gastric cancer xenograft model. Increased tumor accumulation and penetration of TZB in tumor improved the anti-tumor effect of TZB. Accumulation of TZB in the tumor increased by 100% after co-administration of ATV compared with that observed for TZB alone. Anti-tumor activity was measured by circularity and FD analyses to determine the fragmentation of nuclear DNA during co-administration of ATV and TZB. At distances of 0 to 80 μm from the vessel, the circularity significantly decreased by 43% during co-administration of TZB and ATV compared with that observed with the administration of TZB alone. Taken together, these results suggest that ATV may expand the interstitial space, which, thereby, promotes the delivery of Alexa-488-TZB and enhances the therapeutic efficacy of TZB.

Although the enhancement in the accumulation of TZB after administration of CTX was greater than that observed with co-administration of ATV, CTX induced hair loss and cognitive impairment (Figure 1). Abnormal hair growth and alopecia are common side effects of systemic cancer treatment using CTX [33]. However, we showed that ATV did not induce abnormal hair regrowth in mice.

A further advantage of co-administration of ATV could be a decrease in radio-resistance during radio-immunotherapy, which can be induced by IL6 during TZB therapy [34,35]. Co-administration of ATV during radio-immunotherapy using α-particles such as ^211^At and ^225^Ac offers another advantage [36]. α-particles show much higher linear energy transfer (50–230 keV/μm) within a short range in tissues (28–50 μm) than do β-particles emitted by radionuclides such as ^131^I, ^90^Y, or ^177^Lu (LET = 0.1–1.0 keV/μm) whose range is 2 to 10 mm in the tissue [37]. Deep penetration of TZB into a tumor is important to avoid off-target normal tissue toxicity. We found that the depth at which the highest amount of Alexa-488-TZB occurred was shifted from 15 μm to 27 μm when ATV was co-administered (Figure 4E). Therefore, co-administration of ATV may offer an advantage during radioimmunotherapy using α-particles.

Collectively, our results demonstrate that the co-administration of ATV and TZB blocked the development of cognitive impairment. To the best of our knowledge, our study is the first to demonstrate this effect on cognitive impairment during TZB therapy. We also showed that ATV could rescue cognitive impairment during TZB therapy. In addition, we showed that ATV enhanced the therapeutic efficacy of TZB in tumors. This approach represents a promising therapeutic strategy for enhancing TZB treatment without the side effects of cognitive impairment or hair loss.

## 5. Conclusions

We showed ATV could rescue cognitive impairment without hair loss side effect during TZB therapy. In addition, we demonstrated that ATV could enhance the anti-tumor effect of TZB. Our suggested regimen in this study could be translated to patient care in clinics.
